# Topological Darkness in Optical Heterostructures: Prediction and Confirmation

**DOI:** 10.1021/acsphotonics.3c00879

**Published:** 2023-09-12

**Authors:** Emma Cusworth, Vasyl G. Kravets, Alexander N. Grigorenko

**Affiliations:** Department of Physics and Astronomy, University of Manchester, Manchester, M13 9PL, United Kingdom

**Keywords:** topological darkness, topological photonics, zero reflection, phase singularities, dark metamaterials

## Abstract

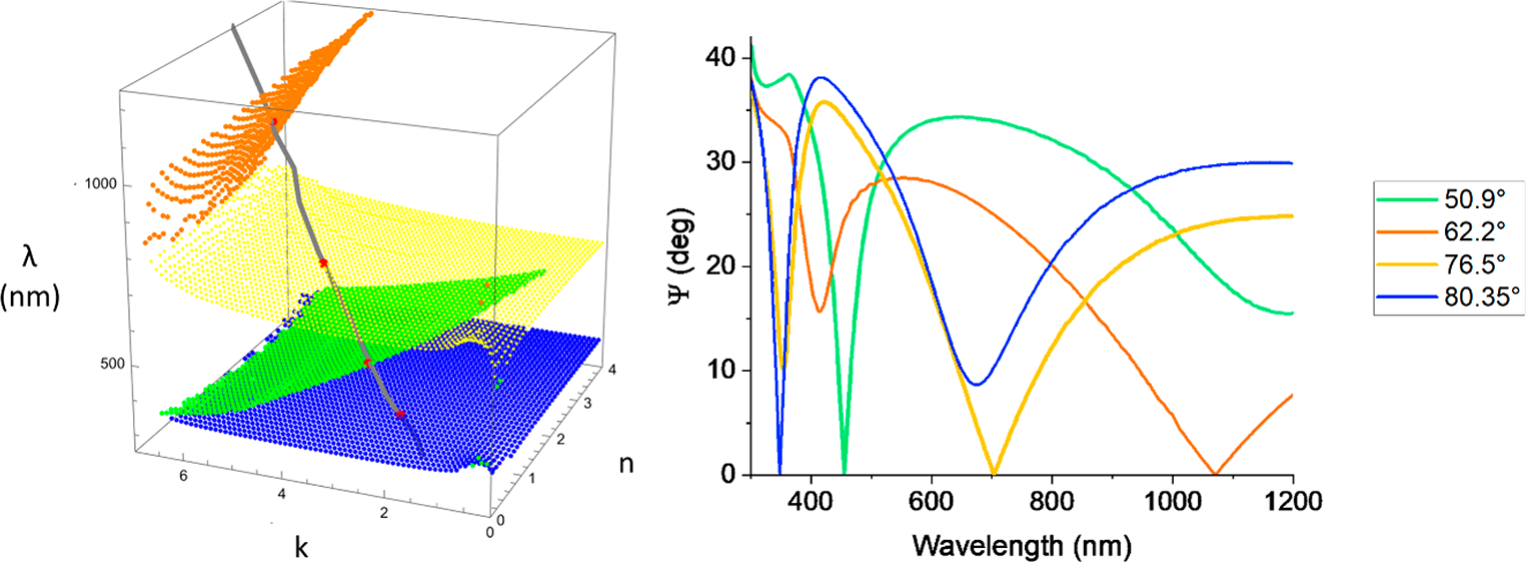

Topological darkness is a new phenomenon that guarantees zero reflection/transmission of light from an optical sample and hence provides topologically nontrivial phase singularities. Here we consider topological darkness in an optical heterostructure that consists of an (unknown) layer placed on a composite substrate and suggest an algorithm that can be used to predict and confirm the presence of topological darkness. The algorithm is based on a combination of optical measurements and the Fresnel equations. We apply this algorithm to ultrathin Pd films fabricated on a Si/SiO_2_/Cr substrate and extract four different points of topological darkness. Our results will be useful for topological photonics and label-free optical biosensing based on phase interrogation.

## Introduction

Topological darkness^1−3^ (TD) provides exactly zero reflection (or transmission) of polarized light from a layered structure. It has the attractive property that, due to topological arguments, the complete suppression of reflected (or transmitted) light happens even in the presence of small imperfections in fabricated structures.^1−3^ TD leads to the Heaviside-like jump of light phase,^4^ topological phase singularities^5^ and could provide topological polarization singularities.^6,7^ Due to extreme phase changes near the points of darkness and robustness of the phenomenon, TD was recently successfully applied to demonstrate ultrasensitive biosensing^1,8−14^ and could become an integral element of topological photonics.^15^

Although TD is a relatively young phenomenon, it is connected to a variety of approaches and methodologies discussed before. One of these is the darkness of light in real space introduced in the studies of optical vortices,^16^ for review see ref (17). (In contrast, TD discusses darkness in Fourier space.) An important example of a system connected to TD is a perfect absorber.^18−21^ However, while a perfect absorber also exhibits zero reflection and spectral phase singularities, it is not clear whether the perfect absorption or phase singularities are topologically protected and could survive fabrication irregularities. Other examples of systems with extremely low reflection include surface plasmon resonance protected by graphene,^22^ optical Tamm states,^12,23^ systems with strong coupling,^24^ and Fabry-Pérot microcavities.^11^

Originally, TD was introduced for a simple system, where a thin film under study is placed on top of a simple substrate. The optical constants of the film were assumed to be known or calculated using a suitable mean-field theory. In this case, the intersections of the spectral dispersion curve with zero reflection surfaces (see below) provide points of TD. In more recent works, however, a film under study (*aka* a top functional layer, a metasurface, etc.) is often made of a nanostructured and/or composite material with unknown optical properties and is placed on a composite substrate that includes several different layer properties of which are known.

The purpose of this work is to propose a simple algorithm that will allow one to predict and confirm the presence of TD in such systems using variable angle optical measurements and the Fresnel equations. To show the effectiveness of our algorithm, we apply it to ultrathin Pd films (3.5 nm) fabricated on a composite Si/SiO_2_/Cr substrate where we predict and confirm four points of topological darkness. Our results will provide wherewithal to tackle TD in composite heterostructures and will be useful for applications in topological photonics and label-free optical biosensing based on phase of light.^4,25,26^

## Results and Discussion

### Principle of Topological Darkness

**Figure 1:**
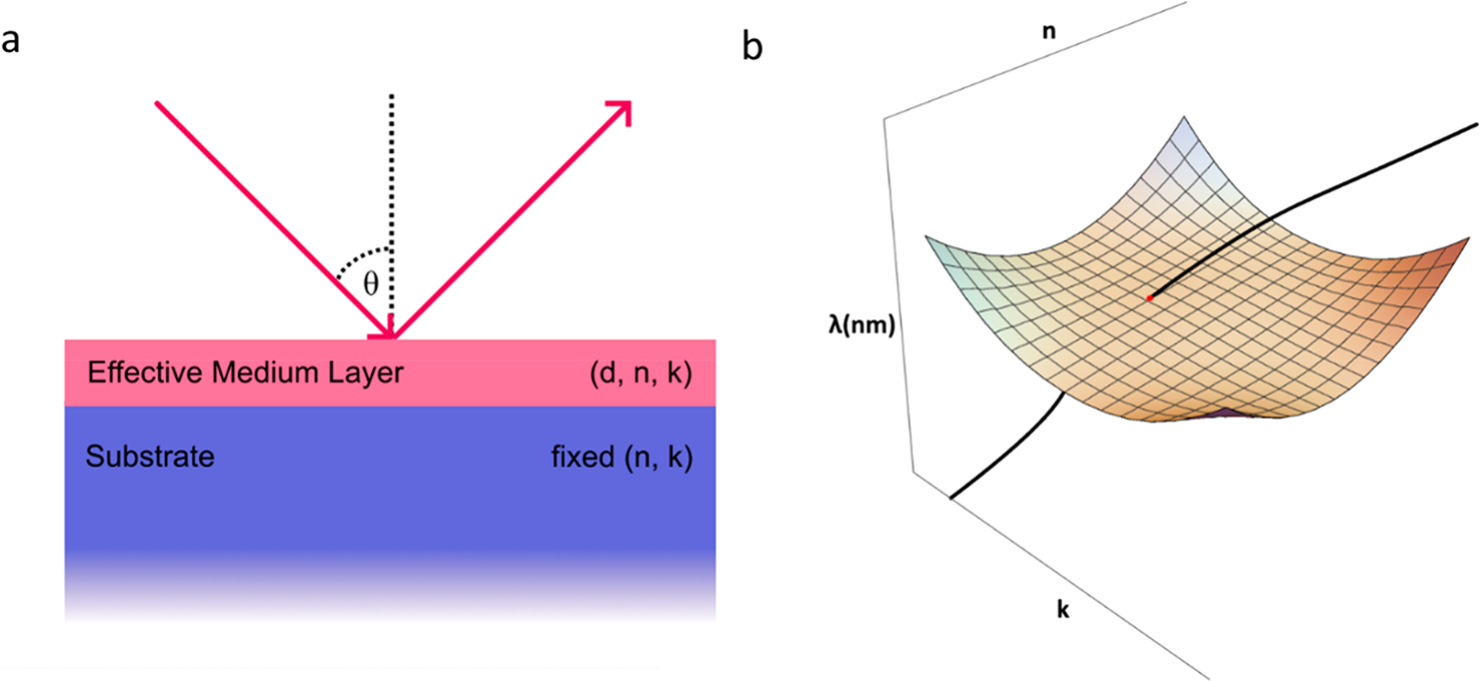
Concept of TD. (a) Schematics of a sample. All of the sample layers have fixed thicknesses. Red arrows show incident and reflected light. (b) The red point of intersection of spectral dispersion curve *n*(λ), *k*(λ) of the top layer with the zero reflection surface (the orange surface with mash) is topologically protected.

Figure 1 briefly reviews the main features of the TD phenomenon.^1−3^ Consider a layer of thickness *d* placed on the top of a substrate, as shown in Figure 1a, with dispersion characteristics *n*(λ), *k*(λ), where *n*(λ) is the real part and *k*(λ) is the imaginary part of the complex refractive index of the layer *N*(λ) = *n*(λ) + *ik*(λ).^27^ (Here, λ is the light wavelength.) We assume that the top layer can be described by effective medium theory, and its dispersion characteristics are known or can be calculated with the help of suitable mean field theory. The optical properties of the substrate are also assumed to be known. Using the Fresnel equations expressed in the form of the transfer-matrix method,^27^ for any given value of *n* and *k* of a top layer, we can calculate the amplitude reflection coefficients *r*_*p*_ and *r*_*s*_ of the whole structure for *p*- or *s*-polarization, respectively. For concreteness, below we will discuss TD in reflection. The amplitude reflection coefficient  of light is a complex number that provide the amplitudes of both in-phase and out-of-phase reflected electric field.^27^ (Here *E*_0_ is the amplitude of incident light and *E*_*r*_ is the complex amplitude of light reflected from the sample.) In the case of the structure shown in Figure 1a, the reflection coefficient is a function of *n*, *k*, θ, and λ: *r* = *r*(*n*,*k*,θ,λ).

Total darkness in reflection happens where the reflection coefficient of the whole structure is exactly zero, *r*(*n*,*k*,θ,λ) = 0. This imposes two conditions on the parameters (as we require that both real and imaginary parts of complex reflection coefficient *r*(*n*,*k*,θ,λ) should be zero simultaneously) and implies that for each given combination of *n* and *k* of the effective medium layer (EML) we can find both wavelength λ(*n*,*k*) and incidence angle θ(*n*,*k*) that provide exactly zero reflection for the structure. Plotted in (*n*, *k*, λ) coordinates, this procedure yields a continuous surface which we refer to as a zero reflection surface (ZRS), Figure 1b. It is worth noting that it is possible to find several zero reflection surfaces corresponding to the same structure as there could be a set of solutions (λ_*i*_(*n*,*k*), θ_*i*_(*n*,*k*)) of the equation *r*(*n*,*k*,θ,λ) = 0 depending on the effective layer film thickness and a range of (*n*, *k*). (Here *i* is the index denoting the solution number.) We then plot the dispersion curve of EML (*n*(λ), *k*(λ), λ) and ZRS calculated for the structure (*n*, *k*, λ(*n*,*k*)) on the same graph; see Figure 1b. When a reasonably smooth dispersion curve of EML starts and ends at the different sides of the zero reflection surface as shown in Figure 1b, it will inevitably intersect ZRS providing a point of total darkness (the absence of reflection) due to the Jordan–Brouwer separation theorem,^28^ which is a higher dimension generalization of the Jordan curve theorem.^29,30^ (The Jordan theorem necessitates that a curve that starts in one region and ends in another intersects with the boundary between them.) Small imperfections in EML could change the dispersion curve; however, they will not change the fact that the dispersion curve starts and ends at the different sides of ZRS leading again to a guaranteed zero reflection point at possibly different wavelength and angle of incidence. This implies that the existence of darkness in such structures is topologically protected by the Jordan–Brouwer theorem and, therefore, this darkness was termed as topological darkness.^1,2^

### Topological Darkness in Composite Optical Heterostructures

Often, optical systems with a top functional layer (*aka* a functional metasurface) are more complicated than the simple system shown in Figure 1a. The top layer can be nanostructured or made of alloys (depending on an application) with unknown optical properties. In addition, the top layer could be fabricated on a composite substrate that allows useful functionalities (e.g., gating of the top layer). Therefore, we can put forward a natural question: can one predict and confirm the presence of TD in such heterostructures by measuring optical reflections of a sample? For the rest of the manuscript, we will quantify sample reflection in terms of the ellipsometric parameters Ψ and Δ defined by the expression:^31^
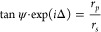
Measurements of ellipsometric parameters are much more accurate than measurements of reflection intensities. It is worth noting that TD in *p*-polarization corresponds to Ψ = 0°, while TD in *s*-polarization corresponds to Ψ = 90°. (At the same time, all of our subsequent considerations could be applied to the case where reflection intensities of *p*- and *s*-polarized light are measured.)

**Figure 2:**
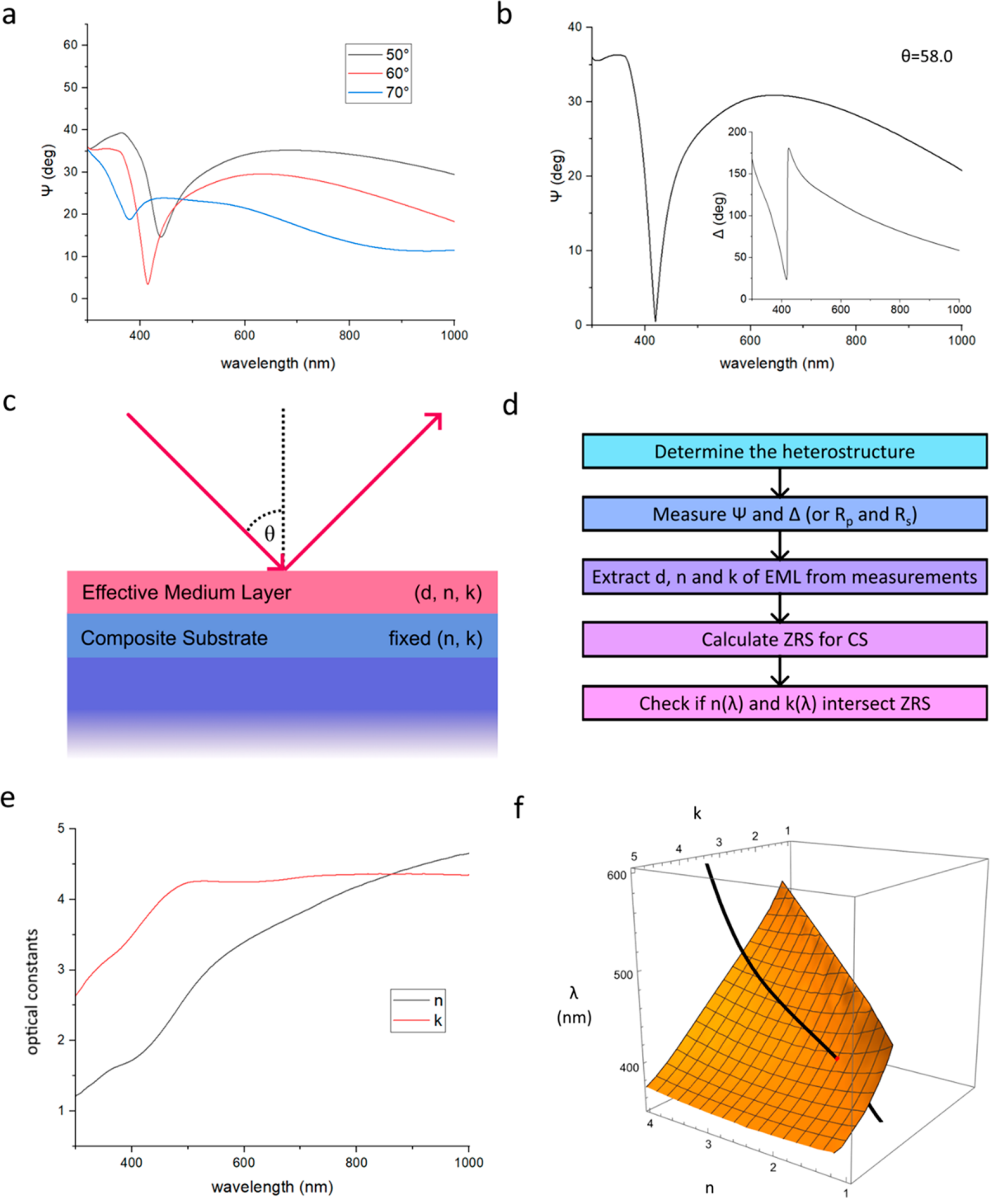
Topological darkness in composite heterostructures. (a) Ellipsometric reflection spectra for different angles of incidence for a 5 nm layer of chromium placed on a composite substrate made of 290 nm of SiO_2_ on Si. (b) Ellipsometric reflection spectra Ψ(λ) observed at an angle of incidence 58° for the same sample. The inset shows the ellipsometric phase Δ behavior. (c) Composite heterostructures discussed in this work. (d) The algorithm of predicting and confirming TD. (e) Optical constants of the top layer extracted from ellipsometry measurements. (f) The red point of intersection of spectral dispersion curve *n*(λ), *k*(λ) of the top layer with the zero-reflection surface (the orange surface with mash), is topologically protected.

As an example, Figure 2a,b provides spectra of Ψ and Δ calculated at different angles for some sample under study. Can we predict whether a sample with the spectra of Figure 2a possesses points of TD? Can we confirm that a reflection spectrum Figure 2b indeed is measured near the TD point with reflection minima at wavelength of ∼420 nm? Here we give a positive answer to both questions. To be precise, we will provide an algorithm for finding points of TD for a composite optical heterostructure shown in Figure 2c. This heterostructure comprises a thin top functional layer with unknown thickness *d* and a composite substrate (CS) that can contain any number of layers with known optical properties and thicknesses. It is assumed that the top layer can be described by effective medium theory and possesses some unknown optical constants (*n*(λ), *k*(λ)). Then, the algorithm of predicting and confirming TD for a heterostructure of Figure 2c is shown in Figure 2d and is as follows:

1. Fix a heterostructure under study. In other words, fix the total number of layers, thicknesses and optical constants of layers in CS. Check whether there exist anisotropic layers in the structure (by either design or fabrication procedures).
2. Perform variable angle spectroscopic measurements of sample reflection. Our reflection data of choice are spectra of ellipsometric parameters measured at different angle of incidence (which is provided by variable angle spectroscopic ellipsometry^31^). However, measurements of reflection intensity of *p*- and *s*- polarization at different angle of incidence are also possible.
3. By fitting measured data using Fresnel theory extract *d*, *n*(λ), *k*(λ) of EML. Most ellipsometers have software that allows one to perform such a fit. Since an ellipsometric spectrum at a given angle of incidence and given wavelength has two measured data (Ψ and Δ), at least two spectra of different angles of incidence are required to extract 3 variables (*d*, *n*(λ), *k*(λ)) of EML at each wavelength. (Actually, even one angle would suffice as thickness *d* is shared by many points of the spectral measurements.) Practically, one needs to measure spectra at 3–4 angles in order to check that the top layer can indeed be described by effective medium theory (EMT). Three important features have to be taken into account during the fit. (A) The thickness of the top layer could be different from target fabrication thickness and needs to be a fit parameter. This is especially important for nanostructured layers, see discussions in refs (32 and 33). (B) It is imperative to check whether the top layer can be described by EMT by performing enough measurements of ellipsometric spectra at different angles of incidence. A decade ago there was a tendency to extract (*n*, *k*) of an unknown thin nanostructured layer from reflection/transmission spectra measured just at normal angle of incidence without performing variable angle measurements. This led to many erroneous claims in cases where optical properties of a nanostructured layer under study did not follow EMT.^32,33^ It is also necessary to check whether a top layer should be described by an isotropic or anisotropic medium by performing fits to the measured reflection data and examining a mean standard error (MSE) of the fit. (C) Finally, one needs to check that the conductivity of the top substrate sublayer does not change the properties of the EML. When a conductive sublayer with sufficient conductivity is present underneath a nanostructured substrate with metallic inclusions, the voltage difference (produced by the electric field of the electromagnetic wave) induces the current between metallic inclusions through the sublayer leading to so-called resistive coupling (or current-induced coupling) of nanoelements.^34,35^ As a result of such coupling, localized plasmon resonances of metallic nanoparticles can be completely suppressed at normal incidence^36^ and restored under larger angles of incidence.^35^ The resistive coupling therefore could impose optical nonlocality into the top layer leading to breaking down its description by EMT.
4. Calculate ZRS for the composite substrate using appropriate Fresnel theory and thickness d extracted from the fit. Assume that the top layer has a given optical constants (*n*, *k*) and calculate wavelength λ(*n*,*k*) and incidence angle θ(*n*,*k*) that provides exactly zero reflection for the whole structure by solving equation *r*(*n*,*k*,θ,λ) = 0. The matrix form of the Fresnel theory seems to be most suited to this task.^27^ As in the case of a simple substrate, the calculation for a composite substrate can yield several different ZRS.
5. Plot the dispersion curve of EML and ZRSs calculated for the structure on the same graph and look for the intersection points which corresponds to TD.

We applied this algorithm to the simulated data shown in Figure 2a,b. The Fresnel fit to the optical data yields the optical constants of the top layer, Figure 2e. Figure 2f plots the dispersion curve for the top layer together with the ZRS calculated for the whole structure. We see that the dispersion curve indeed intersect the ZRS leading to the point of topological darkness at wavelength ∼420 nm and angle of incidence of ∼58° hence confirming the presence of TD for the sample with optical spectra of Figure 2a,b.

### TD in Ultrathin PD Films Fabricated on a Composite Substrate

**Figure 3:**
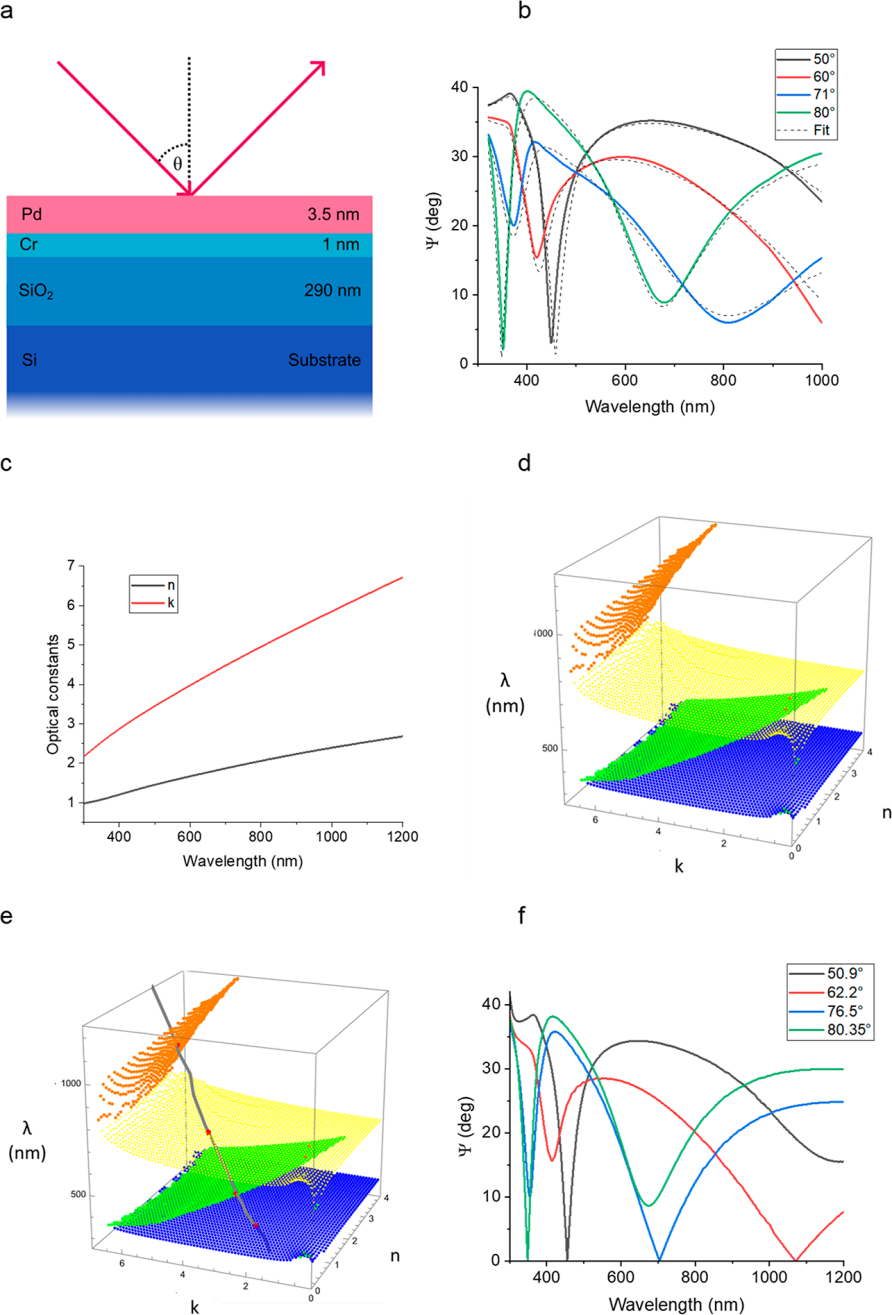
Topological darkness in composite heterostructures: prediction and confirmation. (a) A schematic drawing of the studied sample. Each layer is labeled with its thickness. (b) Measured ellipsometric reflection spectra as a function of incident angle. The dotted lines show the transfer-matrix fitting of the spectra. (c) The optical constant of the Pd film used for fitting. (d) Zero reflection surfaces for the studied structure calculated for *p*-polarized light. (e) The intersection of the dispersion curve of the Pd film (*n*(λ), *k*(λ), and λ) with ZRSs. The red intersection points predict 4 points of TD. (f) The experimental ellipsometric reflection, Ψ, showing 4 points of TD (where Ψ = 0 and, hence, *r*_p_ = 0) observed at the predicted angles of incidence.

We then applied the suggested algorithm of predicting and confirming TD to an experimental heterostructure, as shown in Figure 3a. The structure is to a certain extent analogous to the one that we have already studied in connection with phase singularities and phase topological charges.^5^ Here we concentrate on predicting and confirming the points of TD in this composite heterostructure. The studied layered structure comprised an ultrathin 3.5 nm layer of Pd with a 1–1.5 nm Cr adhesion layer fabricated on the top of a 290 nm layer of SiO_2_ and a Si substrate (Figure 3a). The fabrication of the sample is described in Methods.

First, we measured ellipsometric spectra of the optical reflection of our sample under different angles of incidence, Figure 3b. Second, we fitted the measured Ψ and Δ spectra under a large range of incident angles using the structure shown in Figure 3a and J. A. Woollam Wvase software. Unexpectedly, a high quality fit was achieved by assuming that the Pd film constants are close to smoothed Palik constants measured on bulk Pd.^37^ These optical constants are plotted in Figure 3c. Third, we calculated ZRSs for *p*-polarized light (shown in Figure 3d) as explained in Methods. Finally, following our algorithm, the dispersion curve along with ZRSs was plotted on the same 3D graph, Figure 3e. The graph yielded 4 points of intersection (marked by the dots of the red color) between the dispersion curve of the top layer and ZRSs. Hence, our algorithm predicts four TD points protected by the Jordan–Brouwer theorem in the studied parameter range for the experimental structure of Figure 3a. To check this prediction, we have measured the reflection spectra near the predicted angles and confirmed the presence of four points of exactly zero reflection, as demonstrated in Figure 3f (check the points of Ψ = 0). It is worth noting that the experimental values of the angles and wavelengths for TD were slightly different from the predicted ones, which can be expected due to possible oxidation of Cr and Pd layers film roughness as well as digitization in construction of ZRSs (Methods). These deviations present a good example of topological protection provided by the Jordan–Brouwer theorem. While we do expect that sample imperfections can cause deviations of the predicted locations of TD with the measured ones, darkness in our samples is still guaranteed by topological arguments.

We note that the studied TD points are not connected to the antireflection coating conditions for the SiO_2_/Si structure. This was checked by putting thickness of Pd and Cr films to zero (in the Fresnel model for the structure) and finding large reflection. The TD points are instead connected to the reflection minima of the Fabry–Perot architecture of our composite heterostructure. However, it is difficult to prove using Fabry–Perot theory that the observed minima will correspond to exactly zero reflection. Table 1 lists the measured and predicted wavelengths and angles of the TD points for the studied structure.

**Table 1:** Properties of TD Points for the Structure are Shown in Figure 3a^a^

	experiment, ellipsometry, λ (nm)	theory, intercept, λ (nm)	experiment, ellipsometry, θ (deg)	theory, minimum, θ (deg)
TD1	349	338	80.35	81.2
TD2	455	447	50.9	47.3
TD3	703	681	76.5	76.5
TD4	1070	1015	62.2	61.7

a Wavelengths and angles of incidence of TD points for the measured and calculated data (Methods) are presented.

**Figure 4:**
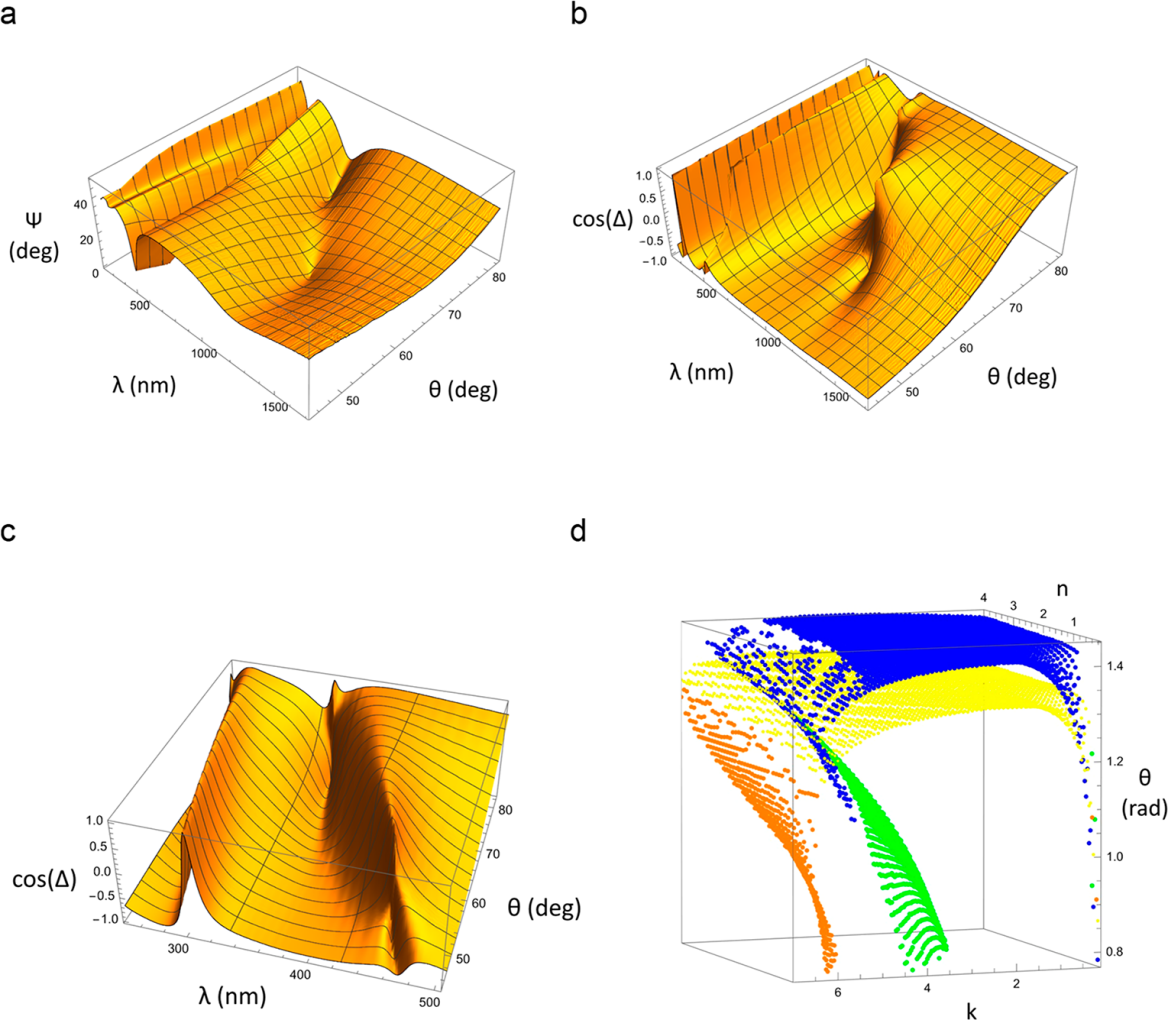
Properties of topological darkness. (a) 3D graph of ellipsometric reflection spectra. (b) cos(Δ) as a function of wavelength and angle of incidence. (c) A zoomed-in part of cos(Δ) as a function of wavelength and angle of incidence. (d) ZRSs in the (*n*, *k*, θ) space.

The points of TD are accompanied by the Heaviside jumps of the phase and phase singularities.^4^ Hence, the presence of TD could also be checked by studying the phase of the light near a TD point. Figure 4 presents the 3D plots of measured Ψ and cos(Δ) to perform this check. (A function cos(Δ) was chosen to show phase in order to avoid the phase ambiguity as phase measurements always result in multiple values separated by 360°. Therefore, Heaviside jumps of the phase at the points of TD correspond to a discontinuous change of cos(Δ) to −cos(Δ).) In Figure 4a we clearly see three zeros in the Ψ plot (one zero is hiding behind the ridge). We also observe four Heaviside-like phase jumps (three in Figure 4b and one in Figure 4c which shows the zoomed and rotated graph of cos(Δ) to have a better view of phase behavior “behind the ridge”).

Finally, we plot ZRSs in the (*n*, *k*, and θ) space in Figure 4d. Examination of the plot suggests that the four ZRSs shown in Figure 4d could actually be produced by two folded ZRSs connected to different modes of the Fabry–Perot cavity. This would explain why the wavelengths of the first two TD points are approximately half of the wavelength of the second two. We believe that investigations of ZRSs for composite heterostructures is fascinating and could bring a lot of surprises.

## Conclusion

We have presented a simple algorithm for predicting and confirming topological darkness for a structure consisting of an unknown functional top layer (a metasurface) placed on top of a composite substrate. The algorithm is based on the combination of optical reflection measurements with the Fresnel equations (in the form of the matrix-transfer method). We describe how the algorithm should be applied and what problems it can encounter. We also demonstrated a practical application of the method to a composite heterostructure, where an ultrathin layer of Pd is fabricated on a substrate that could be used for gating of the top functional layer. Our algorithm revealed multiple zero reflection surfaces and predicted the location of four TD points that were subsequently observed in experiments. The agreement between the predicted and measured properties of the TD points was very good. Our work will allow other researchers to predict the existence of topologically protected zero reflection from composite samples, which could be useful for ultrasensitive biosensing based on the light phase. It is worth stressing again that the property of exactly zero reflection still exists even in the presence of sample imperfections due to topological arguments.

## Methods

### Sample Fabrications

A 1 nm thick Cr adhesion layer and a 3.5 nm thick Pd film were deposited on the top of a 1 mm thick Si substrate covered by SiO_2_ (290 nm) using electron beam evaporation at the base pressure of 1.0 × 10^–6^ Torr. The deposition rate was controlled at 0.1 nm/s. Growth of the metal film was monitored by a calibrated quartz microbalance (CQM) to the accuracy of 0.05 nm.

### Optical Measurements

Optical reflection spectra were measured by a J. A. Woollam M-2000F Variable Angle Spectroscopic Ellipsometer with 245–1690 nm wavelength range, 45–90° angle of incidence range. The light reflection form the samples is described in terms of ellipsometric parameters Ψ and Δ as follows: , where *r*_*p*_ and *r*_*s*_ are complex reflection amplitudes for *p*- and *s*-polarizations, respectively.^31^ The accuracy of measurements of both ellipsometric parameters Ψ and Δ in the M2000F ellipsometer is better than 0.01°. Since ellipsometry measures the ratio of two reflections, variations of light intensity of light source cancel out suppressing the amplitude noise considerably and making ellipsometry an extremely sensitive measuring technique. The light source in the M-2000F is a 75 W Xe arc lamp producing light of a ultraviolet–visible-IR spectrum. In addition to ellipsometric parameters Ψ and Δ, the ellipsometer allowed us to measure separately the intensity reflections for *p* and *s* polarized light *R*_*p*_ and *R*_*s*_, respectively, at various angles of incidence.

### Fresnel Fitting

The Fresnel fitting of the measured optical spectra was performed with the help of a J. A. Woollam Wvase ellipsometric software package based on the transfer-matrix method. The Wvase software allows one to model composite optical heterostructures, has an extensive library of optical constants of various materials, and can deal with anisotropic layers, materials with nontrivial permeability and many special optical materials. It is possible to fit optical constants of several layers to the measured data, if necessary.

### Calculations of ZRS and TD Points

A model of the sample was constructed using the transfer-matrix method using Mathematica. All of the known refractive indices were taken from Palik’s book.^37^ The refractive index of the Pd layer is varied so that a separate set of matrices is constructed for each combination of *n*, *k*, λ, and θ so that values of Ψ can be calculated. Due to the large parameter space, we chose to use a resolution of 0.1 for *n* and *k*; 2 nm for wavelength and 1° for intercept angle. We chose a spectral range of 250 to 1250 nm. We varied *n* from 0.1 to 4 and *k* from 0.1 to 7. We chose an angular range of 45° to 85° so that the entire angular range of the ellipsometer was covered. For each combination of *n*, *k* and θ the minima of Ψ as a function of wavelength was found. These minima are then separated into groups by wavelength. The smallest value of Ψ for each value of *n* and *k* is then found to form a surface for each wavelength group. The angular and spectral resolutions of these surfaces are then improved by constructing additional matrices for values of θ and λ above and below each point on each surface at a resolution of 0.1 nm and 0.1 °Finally, each point with a Ψ value greater than 0.01° were discarded. As a result, an intersection with the surface have an accuracy in a value of Ψ less than 0.01°.

Ordinarily, models of this type find solutions in which Ψ is exactly zero. This is because an algebraic expression for zero Ψ can be determined for each value of *n* and *k*. However, the algebraic approach is computationally intensive. It also makes it difficult to determine how many ZRS are in a given range of parameters. For these reasons, the digital method described above was used instead. Ideally, a hybrid of these two methods could be used in the future to combine their advantages.

For both of these methods, it is difficult to determine the exact location of the intercept between the ZRS and the dispersion curve of the EML due to the limited ZRS resolution in *n* and *k*. In order to overcome this, a second transfer matrix model was developed which reduced the dimensionality of the parameter space, allowing for much improved resolutions in wavelength and intercept angle. The locations of the minima of Ψ are then determined, which are equivalent the location of the ZRS intercepts. Using this simplified method, resolutions of 0.00005° for intercept angle and 0.0005 nm for wavelength were achieved for each TD point, and the calculated Ψ values for these points were all bellow 10^–5^. These improved results are made possible by repeatedly generating new matrices around each TD point at progressively higher resolutions.
